# Development of Parvalbumin-Expressing Basket Terminals in Layer II of the Rat Medial Entorhinal Cortex

**DOI:** 10.1523/ENEURO.0438-17.2018

**Published:** 2018-06-26

**Authors:** Nina Berggaard, Ingvild E. Bjerke, Anna E. B. Paulsen, Linh Hoang, Nan E. T. Skogaker, Menno P. Witter, Johannes J. L. van der Want

**Affiliations:** 1 Department of Clinical and Molecular Medicine, Faculty of Medicine and Health Sciences, NTNU Norwegian University of Science and Technology, Trondheim 7491, Norway; 2 Kavli Institute for Systems Neuroscience, Center for Computational Neuroscience, Egil and Pauline Braathen and Fred Kavli Center for Cortical Microcircuits, NTNU Norwegian University of Science and Technology, Trondheim 7491, Norway

**Keywords:** Developmental Biology, Medial entorhinal cortex, Parvalbumin, Synapse, Ultrastructure

## Abstract

Grid cells in layer II of the medial entorhinal cortex (MEC LII) generate multiple regular firing fields in response to the position and speed of an individual within the environment. They exhibit a protracted postnatal development and, in the adult, show activity differences along the dorsoventral axis (DVA). Evidence suggests parvalbumin-positive (PV^+^) interneurons, most of which are perisomatic-targeting cells, play a crucial role in generation of the hexagonal grid cell activity pattern. We therefore hypothesized that the development and organization of PV^+^ perisomatic terminals in MEC LII reflect the postnatal emergence of the hexagonal firing pattern and dorsoventral differences seen in grid cell activity. We used immuno-electron microscopy to examine the development of PV^+^ perisomatic terminals and their target somata within dorsal and ventral MEC LII in rats of postnatal day (P)10, P15, and P30. We demonstrate that in dorsal and ventral MEC LII, the cross-sectional area of somata and number and density of perisomatic PV^+^ terminals increase between P10 and P15. A simultaneous decrease was observed in cross-sectional area of PV^+^ terminals. Between P15 and P30, both MEC regions showed an increase in PV^+^ terminal size and percentage of PV^+^ terminals containing mitochondria, which may enable grid cell activity to emerge and stabilize. We also report that dorsal somata are larger and apposed by more PV^+^ terminals than ventral somata at all stages, suggesting a protracted maturation in the ventral portion and a possible gradient in soma size and PV^+^ basket innervation along the DVA in the adult.

## Visual Abstract

**Figure:**
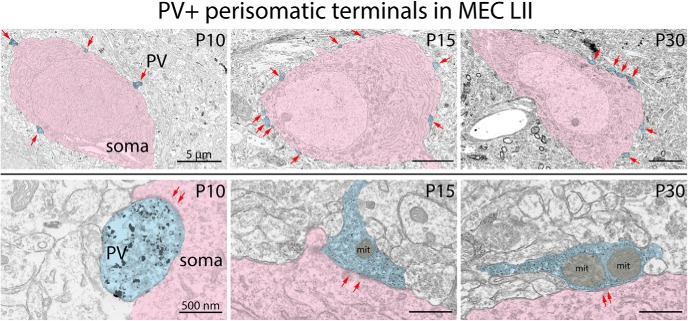

## Significance Statement

Grid cells within layer II of the medial entorhinal cortex produce a regular firing pattern in response to the environment during exploration. This pattern emerges after eye-opening in rodents and exhibits a gradient along the dorsoventral axis. Input from parvalbumin-expressing (PV^+^) interneurons is crucial for maintaining the grid pattern; however, how the PV^+^ innervation develops is unclear. Here we examine the structural development of dorsal and ventral perisomatic PV^+^ terminals and target somata. We show that the size of grid-related somata and number of PV^+^ terminals increase before eye-opening, whereas PV^+^ terminals increase in size during grid activity stabilization. We further demonstrate that maturation in the ventral part is delayed, and that there are dorsoventral differences in young adults.

## Introduction

The hippocampal formation comprises several strongly connected brain structures containing cell types with distinct activity patterns that together form the internal representation of space (Moser et al., 2008). Central to this spatial system is layer II of the medial entorhinal cortex (MEC LII), the location of most of the spatially modulated grid cells. Dependent on the position and speed of an individual, grid cells produce hexagonally arranged firing fields that form a matrix of the environment (Hafting et al., 2005). To attain the sharp onset and end of these “grid fields,” grid cells require tight inhibitory control with high temporal precision by presynaptic neurons (Miao et al., 2017).

The morphologic identity of grid cells is controversial, and knowledge about synaptic connectivity in MEC LII is limited. It has been found that the MEC LII microcircuitry is strikingly inhibitory, with few direct excitatory connections between principal cells (Couey et al., 2013; Pastoll et al., 2013; Fuchs et al., 2015). Supporting evidence indicates that the most common type of inhibitory cell, the parvalbumin expressing (PV^+^) interneuron, provides strong regulation of grid cells through monosynaptic inputs (Buetfering et al., 2014; Miao et al., 2017). PV^+^ interneurons are fast-spiking and GABAergic, and although PV^+^ cells comprise several subtypes, the majority are basket cells targeting perisomatic regions (Baude et al., 2007).

Grid cell activity has a specific timeline of maturation and displays dorsoventral differences. In rodents, for instance, grid cells do not start to exhibit a stable hexagonal activity pattern before postnatal day 16 (P16). However, after this time point, there is a rapid increase in number of mature grid cells, reaching close to adult levels around P22 (Langston et al., 2010; Wills et al., 2010, 2012). Electrophysiological recordings have demonstrated that the size of grid fields and the distance between them increases along the dorsoventral axis (DVA) of MEC LII (Brun et al., 2008; Stensola et al., 2012). These spatiotemporal differences in grid cell activity likely reflect differences in connectivity within MEC LII. Interestingly, concomitant with the increase in grid field size and spacing, there is a decrease in overall number of PV^+^ terminals in MEC LII along the DVA, although the number of GABAergic terminals remains constant (Beed et al., 2013). Moreover, the DVA exhibits a gradient in maturation, with dorsal neurons developing before ventral ones (Ray and Brecht, 2016; Donato et al., 2017). Considering the putative involvement of PV^+^ basket cells in modulating grid cell activity, we hypothesized that synaptic innervation by PV^+^ basket cells would display changes during postnatal development and along the DVA that parallel the spatiotemporal differences in grid cell activity. We used immuno-electron microscopy to investigate and perform quantitative analysis of the structural maturation of cell somata and PV^+^ basket cell terminals apposing these somata in the dorsal and ventral portions of MEC LII.

## Materials and Methods

### Animals and tissue processing

Five male Long-Evans rats provided by the Norwegian University of Science and Technology were studied from P10 (two rats), P15 (one rat), and P30 (two rats). All animal procedures were performed in accordance with the Norwegian University of Science and Technology animal care committee’s regulations. The rats lived in a controlled environment with lights on from 8:00 pm to 8:00 am, and with food and water available *ad libitum*. P30 rats were anaesthetized with 4%–5% isofluorane in 1 liter O_2_/min with a subsequent overdose of pentobarbital, and the rats at P10 and P15 were kept anaesthetized with 0.5 liter O_2_/min. When the absence of reflexes was confirmed, transcardial perfusion was performed with fresh Ringer’s solution comprising 145 mm NaCl, 3 mm KCl, and 2 mm NaHCO_4_. The pH of the solution was adjusted to 6.9. The perfusion fluid was changed to cold 4% paraformaldehyde (PFA; Merck) and 0.1% glutaraldehyde (GA; Merck) in 0.1 m PBS. Brains were removed from the skull, postfixed in the same solution at 4˚C for at least 12 h, and stored in 0.1 m PBS until further processing. All perfusions were performed at noon ± 2 h.

**Figure 1.**
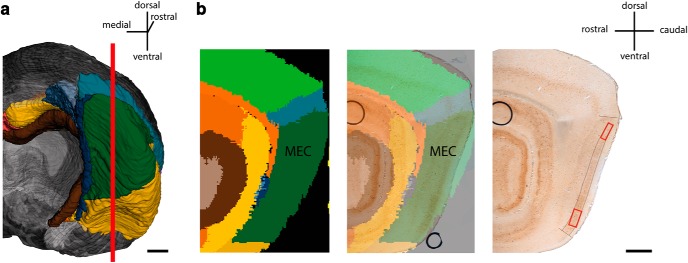
Position of regions of interest used for immuno-electron microscopy. ***a,*** Caudal view of rat brain, right hemisphere, without cerebellum, made by using the Waxholm Space Atlas (Kjonigsen et al., 2015; Papp et al., 2014). Red line signifies cutting plane, showing region from which parasagittal sections used in the study were taken. ***a,*** Example of parasagittal section of the caudal part of rat brain. Left: Waxholm Space Atlas representation showing subdivisions. Middle: Waxholm Space Atlas representation superimposed onto PV^+^ immunolabeled section used in study. Right: A corresponding PV^+^ immunolabeled vibratome section. Black lines delineate MEC LII. Red boxes show the dorsal and ventral positions from which the tissue was processed for further analysis. Scale bars: 1 mm.

The brains were rinsed in cold 125-mm PBS, and 60-µm-thick parasagittal sections were cut using a vibratome (Leica VT1000S). Sections containing the region of interest were collected and postfixed in 4% PFA and 0.1% GA in 0.1 m PBS for 2 h before being stored at 4˚C in 125 mm PBS. The anatomic location of the vibratome sections was determined by spatial registration of section images to the Waxholm Space atlas of the rat brain (Papp et al., 2014; Kjonigsen et al., 2015) using the software tool QuickNII (Puchades et al., 2017). The vibratome sections selected for electron microscopy were mapped to the lateral part of the temporal lobe at atlas levels 375–380 (corresponding to a distance of 190 µm in the atlas), and the locations of the blocks taken for electron microscopy were thus confirmed to be situated within the MEC (Fig. 1*a*,*b*).

### Immuno-electron microscopy

Sections were rinsed in 0.1 m phosphate buffer (PB) and incubated in 0.1 m PB + 0.05% Triton X-100 (0.05% PB-TrX) for 20 min. After rinsing in 0.1 m PB, endogenous peroxidase activity was blocked with 1% H_2_O_2_ and 50% methanol in 0.1 m PB for 30 min. Sections were then rinsed and incubated in a solution of 1% BSA (Sigma-Aldrich, A7034), 1% normal horse serum (NHS, Swant, H0146) and 0.05% PB-TrX for 1–1.5 h. Because of the more fragile tissue, Triton X-100 was not included in the PB buffer for P10 rats. Next, sections were incubated overnight at 4˚C with polyclonal goat anti-parvalbumin **(**Swant, PVG 214 RRID:AB_10000345**)** diluted 1:40,000 in 1% BSA and 1% NHS. The following day, the sections were rinsed and incubated in biotinylated horse anti-goat IgG (Vector Laboratories, BA-9500, also BA9500 RRID:AB_2336123) at 1:400 for 2 h at room temperature. After rinsing, sections were incubated with avidin-biotin-peroxidase (Vectastain ABC kit standard, Vector Laboratories, PK-4000 RRID:AB_2336818) in 0.1 m PB for 1 h at room temperature, with subsequent rinsing in 0.1 m PB and staining in the dark with filtered 0.03% 2,3-diaminobenzidine (DAB; Sigma-Aldrich) and 0.01% H_2_O_2_ in 0.1 m PB. When labeled cells were clearly visible, the reaction was stopped with another round of rinsing in buffer. After microscopic inspection, images were taken for later reference. Glassware washed in a hydrochloric acid solution was hereafter used during the subsequent silver development and gold toning, and all solutions were diluted with Milli-Q water. After rinsing in 2% sodium acetate (Sigma, S8750), sections were kept in 10% sodium thioglycolate (Sigma, T0632) overnight at 4˚C. Sections were then rinsed in 2% sodium acetate. Immunoreactivity was enhanced according to the gold-substituted silver peroxidase method (van den Pol and Gorcs, 1986). In short, after rinsing in 2% sodium acetate, sections were incubated in the freshly prepared silver development solution for 7–8 min, after which the silver development was stopped by a 2-min rinse in 1% acetic acid. Sections were then rinsed in sodium acetate, followed by a 15-min incubation in 0.05% gold chloride. After another rinse in sodium acetate, sections were incubated in 3% sodium thiosulfate and rinsed in sodium acetate.

### Postfixation and embedding

Sections were rinsed in 0.1 m cacodylate buffer and postfixed in 1% OsO_4_ and 1.5% K_4_Fe(CN) _6_ in 0.1 m cacodylate for 15 min. Another rinse in 0.1 m cacodylate buffer ensued before the sections were dehydrated in an ascending concentration of ethanol and embedded in epoxy resin (LX 112, Ladd Research Industries) according to standard protocols. Sections were flat-embedded between two Aclar sheets at 60˚C overnight. Regions of interest along the dorsoventral axis of MEC LII were carefully cut out and glued with epoxy resin onto polymerized epoxy stubs (Fig. 1*b*). After polymerization, ultrathin sections of MEC LII were cut using a Leica UC6 Ultramicrotome. Thin sections were collected on 200-mesh thin-bar copper grids and contrasted with 4% uranyl acetate and 1% lead citrate. Sections were inspected with a transmission electron microscope (JEOL JEM-1011) and imaged using a digital Morada camera.

### Measurements of perisomatic PV^+^ terminals and PV^–^ somata

Selected samples of entorhinal cortex were taken from at least two different 60-µm vibratome sections per age group to obtain independent measurements. To target principal cells of LII, care was taken to select PV-immunonegative somata from deep to superficial LII. To be included for the measurements, cells had to contain a nucleus, and the perisomatic PV^+^ terminals had to display immunolabeling. The synaptic membrane had to be rigidly apposed to the somatic membrane and contain electron dense pre- and postsynaptic specializations exhibited as a membrane widening. Because of the immunolabeling treatment with DAB and silver-gold precipitate, vesicles were not always visible. The cross-sectional area of somata and PV^+^ perisomatic terminals was measured, and the percentage of cross-sectional somata apposed by PV^+^ terminals was calculated. Somata not apposed by PV^+^ terminals were excluded to find the average number of PV^+^ terminals forming perisomatic synaptic contacts per soma and when calculating the density of PV^+^ terminal apposition, which was done by dividing the total length of PV^+^ terminal membrane apposed to a soma by the soma perimeter and multiplying by 100. Finally, the number of perisomatic PV^+^ terminals containing mitochondria was divided by the total number of perisomatic PV^+^ terminals and multiplied by 100 to get the percentage of PV^+^ terminals containing mitochondria. All measurements were performed using iTEM software (Olympus 5.0)

### Statistical analysis

As the cross-sectional area of somata in the ventral part of MEC LII showed a homogeneous variance and normal distribution, the differences over time were assessed with one-way ANOVA and Tukey’s *post hoc* test. The cross-sectional area of somata in the dorsal MEC LII did not display a homogeneous variance; therefore, Welch ANOVA and Games–Howell *post hoc* tests were used to assess developmental differences. An independent *t* test was used to evaluate differences between dorsal and ventral MEC LII and between animals of same age group. Statistical differences in the percentage of cross-sectional somata apposed by PV^+^ terminals and percentage of PV^+^ terminals containing mitochondria were determined by Pearson χ^2^ tests. Because all other variables lacked a normal distribution and homogeneous variance, Kruskal–Wallis *H* tests and Mann–Whitney *U* tests were performed to assess developmental differences, whereas differences between dorsal and ventral regions and between animals of same age groups were examined using Mann–Whitney *U* tests. All statistics were performed using IBM SPSS version 25.0.0.1, and statistical significance was set to *p* ≤ 0.05.

## Results

**Figure 2.**
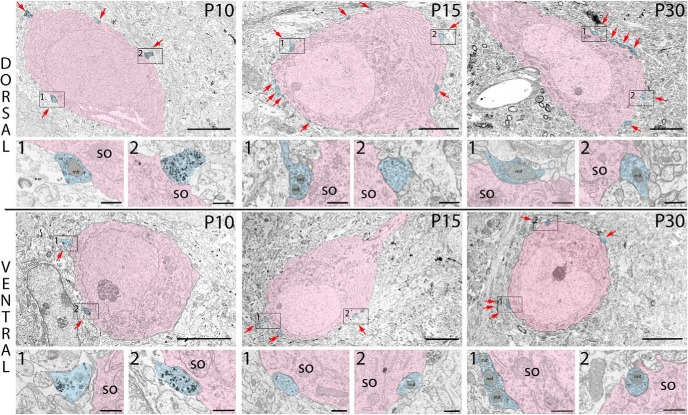
Typical neuronal somata within the dorsal and ventral portion of MEC LII at P10, P15, and P30. PV^+^ terminals (blue) forming synaptic contact onto somata (pink) are indicated by red arrows. The boxes indicate PV^+^ terminals shown in the enlarged figures just below the low-magnification images of the somata. Note that the ventral soma at P10 is apposed by only one PV^+^ terminal; thus, the first close-up image is of a PV^+^ labeled profile in the vicinity. so, soma; mit, mitochondria. Scale bars: images of somata, 5 µm; images of PV^+^ terminals, 500 nm.

We investigated the development of PV^+^ basket cell–related network within MEC LII by measuring the cross-sectional area of PV immunonegative somata and counting the number of perisomatic PV^+^ terminals (hereafter simply referred to as PV^+^ terminals) in dorsal and ventral LII of rats at P10 (*n* = 2), P15 (*n* = 1), and P30 (*n* = 2). Cell somata in layer II were spherical, ovoid, or elongated in shape and less frequently classified as multipolar or fusiform. Somata in the plane of sectioning including a nucleus were used for measurements, assuming that this would give a midcentral representation of the terminal input on that soma. At all three postnatal stages, but more frequently at P15 and P30, cells were apposed by PV^+^ terminals, which formed symmetric synapses and which contained varying degrees of labeling (Fig. 2).

### Soma cross-sectional area

**Figure 3.**
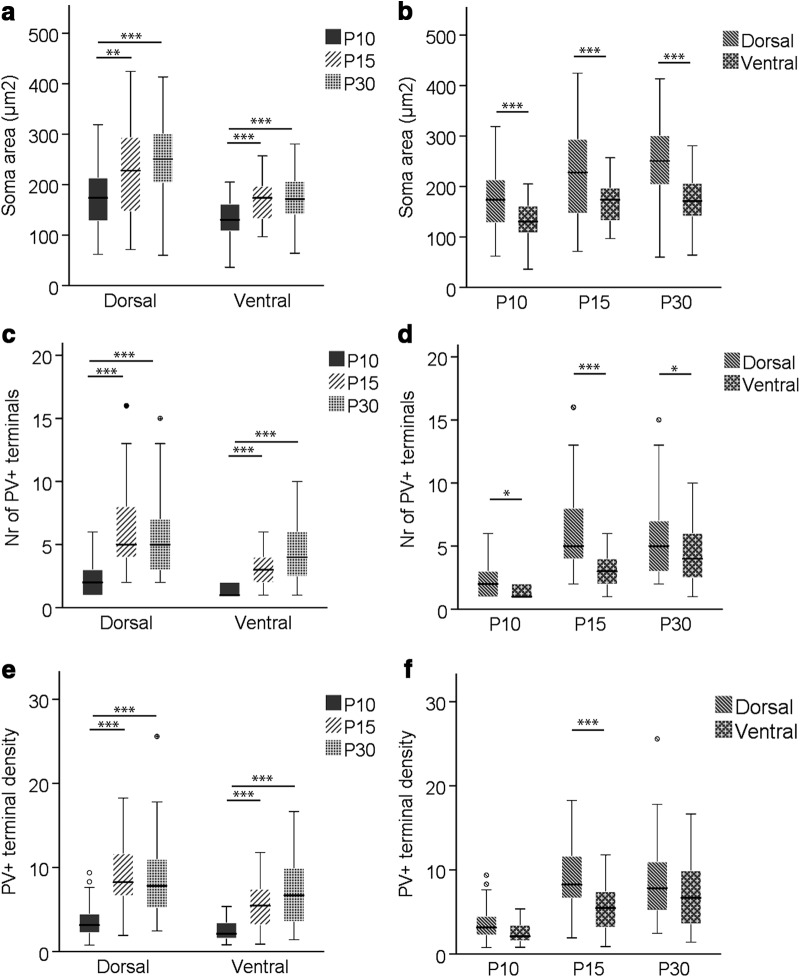
Box-and-whisker plots showing cross-sectional area of somata and number and density of PV^+^ perisomatic terminals per cell per thin section at P10, P15, and P30 in dorsal and ventral MEC LII. ***a, c, e,*** Changes in average cross-sectional soma area and number and density of PV^+^ terminals, respectively, during ontogeny in dorsal and ventral LII. ***b, d, f,*** Dorsoventral differences in average cross-sectional soma area and number and density of PV^+^ terminals, respectively, per time point. The middle bar of each box plot represents the population median, and the 25th and 75th percentiles are represented by the lower and upper boundaries of the boxes, respectively. The minimum and maximum values are represented by the lower and upper ends of the whiskers. Dots represent outliers, i.e., values that exceeded the 75th percentile + 1.5 × (75th percentile – 25th percentile). *, *p* ≤ 0.05; **, *p* ≤ 0.01; ***, *p* ≤ 0.001.

Dorsal and ventral cells in MEC LII displayed changes in cross-sectional soma area during development, as determined by Welch ANOVA (*F*_(2,83.524)_ = 14.512, *p* < 0.001) and one-way ANOVA (*F*_(2,136)_ = 12.422, *p* < 0.001), respectively. The largest increment occurred between P10 and P15, with a 30.9% increase in cross-sectional soma area in dorsal LII (*p* = 0.010, Games–Howell *post hoc* test) and a 27.3% increase in ventral LII (*p* < 0.001, Tukey’s *post hoc* test). The cross-sectional soma area did not increase significantly between P15 and P30, although there was still a slightly larger growth in the dorsal part (12.1%) compared to the ventral one (2.6%; Table 1; Fig. 3*a*). The cross-sectional area of dorsal somata was consistently larger compared with ventral somata, either 31.1% (P10, *t*(62.9) = 3.52, *p* = 0.001, independent *t* test), 34.8% (P15, *t*(56.8) = 3.72, *p* < 0.001, independent t test), or 47.2% (P30, *t*(76.0) = 6.04, *p* < 0.001, independent t test; Table 1; Fig. 3*b*).

**Table 1.** Cross-sectional soma area, percentage of soma sections with PV^+^ terminal apposition, number of PV^+^ perisomatic terminals per cell per thin section, and PV^+^ terminal density at P10, P15, and P30 in dorsal and ventral MEC LII

	Mean ± SEM or % (*n* cells)	
Age, postnatal days	Dorsal	Ventral
Cross-sectional area of somata (µm^2^)		
10	172.94 ± 10.03 (39)	131.91 ± 5.94 (47)
15	226.35 ± 14.56 (44)	167.89 ± 5.90 (46)
30	253.62 ± 11.49 (47)	172.29 ± 7.04 (46)
Soma cross-sections with PV^+^ terminal apposition		
10	82.1 (39)	38.3 (47)
15	100 (44)	95.6 (46)
30	100 (47)	95.6 (46)
Number of PV^+^ perisomatic terminals		
10	2.22 ± 0.28 (32)	1.28 ± 0.11 (18)
15	6.34 ± 0.52 (44)	3.25 ± 0.22 (44)
30	5.74 ± 0.44 (47)	4.23 ± 0.36 (44)
PV^+^ terminal density (%)		
10	3.64 ± 0.38 (32)	2.58 ± 0.30 (18)
15	8.93 ± 0.62 (44)	5.50 ± 0.41 (44)
30	8.71 ± 0.70 (47)	6.97 ± 0.60 (44)

PV^+^ terminal density was calculated by dividing the total length of PV^+^ terminal membrane apposed to a given cross-sectional soma by the perimeter of that soma and multiplying by 100.

### Number and density of PV^+^ terminals

At P10, and to a larger extent in the ventral portion of MEC LII (χ^2^(1) = 16.765, *p* < 0.001), cross-sectional somata absent of PV^+^ terminal apposition were frequently observed. Between P10 and P15, there was an increase in percentage of cross-sectional somata apposed by PV^+^ terminals in the dorsal (82.1% to 100%, χ^2^(1) = 8.625, *p* = 0.003) and ventral (38.3% to 95.6%, χ^2^(1) = 34.413, *p* < 0.001; Table 1) portion. To measure the average number and density of PV^+^ terminals per cross-sectional soma, only somata apposed by PV^+^ terminals were included. Kruskal–Wallis *H* tests revealed statistically significant changes during development in the number of PV^+^ terminals per cross-sectional soma in dorsal (χ^2^(2) = 43.157, *p* < 0.001) and ventral (χ^2^(2) = 30.346, *p* < 0.001) LII. The largest increase was between P10 and P15 in dorsal (185.6%, *U* = 143, *p* < 0.001, Mann–Whitney *U* test) and ventral (153.9%, *U* = 82.5, *p* < 0.001, Mann–Whitney *U* test) part of MEC LII. Between P15 and P30, the dorsal and ventral MEC LII showed opposing trends, with a slight decrease in the dorsal part (–9.5%) and an increase (30.2%) in the ventral part (Table 1; Fig. 3*c*). The number of PV^+^ terminals per cross-sectional soma in dorsal LII was significantly higher than in ventral LII at all three time points (P10: *U* = 181.0, *p* = 0.017; P15; *U* = 393.5, *p* < 0.001; P30: *U* = 743.5, *p* = 0.02, Mann–Whitney *U* test; Table 1; Fig. 3*d*).

Regarding the density of PV^+^ terminals, calculated by dividing the total length of PV^+^ terminal membrane apposed to a given soma by the perimeter of that soma and multiplying by 100, we noticed an ontogenetic change similar to that of the mean number of PV^+^ terminals (dorsal, χ^2^(2) = 38.645, *p* < 0.001; ventral, χ^2^(2) = 22.543, *p* < 0.001, Kruskal–Wallis *H* test), with a statistically significant increase between P10 and P15 in dorsal (145.3%, *U* = 179, *p* < 0.001, Mann–Whitney *U* test) and ventral (113.2%, *U* = 142, *p* < 0.001, Mann–Whitney *U* test) LII. Neither dorsal nor ventral LII showed significant changes between P15 and P30 in PV^+^ terminal density (Table 1; Fig. 3*e*). Somata in the dorsal part of LII had significantly larger PV^+^ terminal density than ventral somata at P15 (*U* = 478, *p* < 0.001, Mann–Whitney *U* test), but not at P10 (*U* = 210, *p* = 0.115, Mann–Whitney *U* test) and P30 (*U* = 823, *p* = 0.094, Mann–Whitney *U* test; Table 1; Fig. 3*f*).

### Maturation of PV^+^ terminals

**Figure 4.**
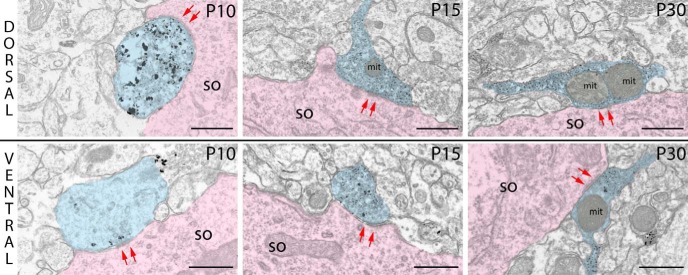
Cross-sections of typical PV^+^ terminals in dorsal and ventral part of MEC LII at P10, P15, and P30. Arrows (red) indicate synaptic contacts between PV^+^ terminals (blue) and somata (pink). so, soma; mit, mitochondria. Scale bars: 500 nm.

To investigate the maturation process of PV^+^ basket innervation, we measured the cross-sectional area of terminals and calculated the percentage of PV^+^ terminals containing mitochondria within dorsal and ventral MEC LII at P10, P15, and P30. Cross-sections of typical terminals found in the dorsal and ventral MEC are shown in Fig. 4.

**Figure 5.**
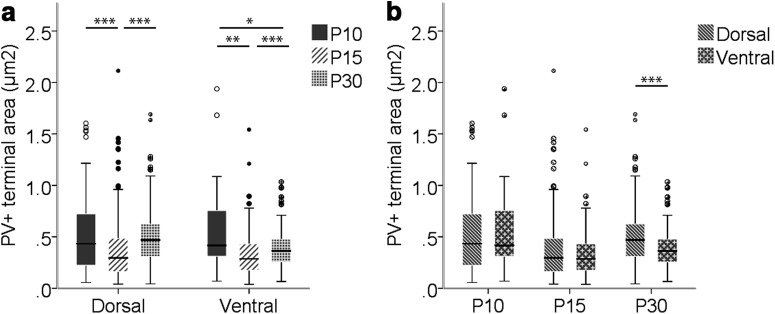
Box-and-whisker plots of the cross-sectional area of PV^+^ terminals at P10, P15, and P30 in dorsal and ventral MEC LII. ***a,*** Changes during development of the cross-sectional area of PV^+^ terminals in dorsal and ventral MEC LII. ***b,*** Dorsoventral differences per time point. The middle bar of each box plot represents the population median, and the 25th and 75th percentiles are represented by the lower and upper boundaries of the boxes, respectively. The minimum and maximum values are represented by the lower and upper ends of the whiskers. Dots represent outliers, i.e., values that exceeded the 75th percentile + 1.5 × (75th percentile – 25th percentile). *, p ≤ 0.05; **, p ≤ 0.01; ***, p ≤ 0.001.

The cross-sectional area of PV^+^ terminals showed significant differences between groups in both dorsal (χ^2^(2) = 49.776, *p* < 0.001) and ventral (χ^2^(2) = 18.794, *p* < 0.001) MEC LII, as determined by Kruskal–Wallis *H* tests. On average, the cross-sectional area of PV^+^ terminals was largest at P10 in dorsal and ventral parts of LII, with a significant decrease in cross-sectional area until P15 (dorsal: –30.8%, *U* = 10,743, *p* < 0.001; ventral: –45.8%, *U* = 1020, *p* = 0.002, Mann–Whitney *U* test). In both regions of LII, this decrease was followed by a significant increase in area of PV^+^ terminals between P15 and P30 (dorsal, *U* = 20456, *p* < 0.001; ventral, *U* = 11276, *p* < 0.001, Mann–Whitney *U* test), such that in the dorsal portion, PV^+^ terminals at P30 and P10 were of approximately the same size. For the ventral portion, however, PV^+^ terminals were significantly smaller at P30 than at P10 (*U* = 1663, *p* = 0.043, Mann–Whitney *U* test; Table 2; Fig. 5*a*). The cross-sectional area of PV^+^ terminals was similar in dorsal and ventral parts of LII at P10 and P15. However, the smaller relative increase in cross-sectional area of ventral PV^+^ terminals than of dorsal PV^+^ terminals between P15 and P30 caused a significant difference in PV^+^ terminal size between dorsal and ventral MEC LII at P30 (*U* = 15259, *p* < 0.001, Mann–Whitney *U* test; Table 2; Fig. 5*b*).

**Table 2.** Cross-sectional area of PV^+^ terminals and percentage of PV^+^ terminals containing mitochondria at P10, P15, and P30 in dorsal and ventral MEC LII

Age, postnatal days	Mean ± SEM or % (*n* terminals)	
	Dorsal	Ventral
Cross-sectional area of PV^+^ perisomatic terminals (µm^2^)		
10	0.52 ± 0.04 (98)	0.59 ± 0.10 (23)
15	0.36 ± 0.02 (294)	0.32 ± 0.02 (149)
30	0.51 ± 0.02 (217)	0.38 ± 0.01 (195)
PV^+^ terminals containing mitochondria		
10	36.7 (98)	30.4 (23)
15	39.1 (294)	30.9 (149)
30	65.4 (217)	63.6 (195)

The percentage of PV^+^ terminals containing mitochondria remained at 35%–40%, and 30%–31% between P10 and P15 in the dorsal and ventral part of LII, respectively. Between P15 and P30, however, there was an increase to 65.4% in dorsal LII (χ^2^(1) = 34.602, *p* < 0.001, Pearson χ^2^ test) and 63.6% in ventral LII until P30 (χ^2^(1) = 36.169, *p* < 0.001, Pearson χ^2^ test). The percentage of PV^+^ terminals containing mitochondria was not significantly different between dorsal and ventral regions of MEC LII between P10 and P30 (Table 2).

To examine whether the results for developmental changes or dorsoventral differences could be affected by potential differences between animals of the same age group, we compared the means between the individual P10 and individual P30 animals for all variables. This was done by using either independent *t* tests or, in the absence of homogeneous variances, nonparametric Mann–Whitney *U* tests. Only variables that were significantly different between animals of the same age group are shown (Table 3). Significant differences were found between the two P30 animals, as one animal had 33.4% fewer PV^+^ terminals per soma per thin section than the other animal. These terminals were in addition 12.8% larger than those of the second animal. Additional differences were found between P10 animals, as ventral cells of one animal were on average 37.1% larger than those of the second animal.

**Table 3.** Statistically significant differences in PV^+^ terminals and somata between animals of same age group

			Mean ± SEM (*n*)		
Variable	Age, days	Region of MEC LII	Animal 1	Animal 2	Test statistics and *p* value
Soma cross-sectional area (µm^2^)	10	Ventral	152.06 ± 6.68 (24 cells)	110.88 ± 7.91 (23 cells)	*t*(45) = 3.99, *p* < 0.001
Number of PV^+^ terminals	30	Dorsal	4.74 ± 0.43 (27 cells)	7.10 ± 0.78 (20 cells)	*U* = 160, *p* = 0.017
PV^+^ terminal cross-sectional area (µm^2^)	30	Dorsal	0.53 ± 0.28 (128 terminals)	0.47 ± 0.25 (89 terminals)	*U* = 4781, *p* **=** 0.044

The differences between individual animals of the same age group are shown, along with associated *p* values derived from independent *t* test (soma cross-sectional area) or Mann–Whitney *U* test (all other variables).

## Discussion

A crucial component for maintaining the spatial tuning of grid cells is the strong inhibitory input they receive by PV^+^ interneurons (Buetfering et al., 2014; Miao et al., 2017). To investigate the structural postnatal development of PV^+^ basket cell perisomatic connectivity and targeted somata within the dorsal and ventral part of MEC LII, we selectively labeled vibratome sections from rats at P10, P15, and P30 with PV antibody and processed them for electron microscopy. We report that the development between P10 and P30 in dorsal and ventral MEC LII follows a similar course, with an increase in soma cross-sectional area and number and density of PV^+^ terminals occurring between P10 and P15. This increase in number and density of PV^+^ terminals occurs in parallel with a reduction in average cross-sectional area of the PV^+^ terminals. Between P15 and P30, PV^+^ terminals show an increase in cross-sectional area and percentage containing mitochondria. We also report that dorsal somata of layer II principal neurons are larger and apposed by more PV^+^ terminals than ventral somata at all developmental stages.

### PV^+^ innervation of principal cells in MEC LII

At P10, and especially in the ventral portion of MEC LII, we found somata that were not contacted by PV^+^ terminals. However, at P15 and P30, PV^+^ terminals formed symmetric synapses with almost all somata, in line with a previous immuno-electron microscopy study in the adult rat MEC (Wouterlood et al., 1995). Although we did not selectively identify somata, a substantial portion of them likely includes principal cells of MEC LII—stellate and pyramidal cells—as they account for ∼65%-67% and 17% of the total cell population in the rat MEC LII, respectively (Gatome et al., 2010). Both cell types have previously been found to exhibit grid cell properties (Tang et al., 2014; Sun et al., 2015). Stellate and pyramidal cells can be identified based on their respective expression of reelin and calbindin, and recent studies have found that the majority of layer II principal neurons are strongly innervated by perisomatic PV^+^ terminals (Armstrong et al., 2016). PV^+^ inhibition of pyramidal cells is, however, controversial. In a recent study, principal cells were classified as stellate, intermediate stellate, pyramidal, and intermediate pyramidal cells, and functional connectivity was observed between PV^+^ cells and all principal cell types except pyramidal cells (Fuchs et al., 2015). This would imply that pyramidal and stellate cells are embedded in completely different inhibitory microcircuits (Witter et al., 2017). A minority of somata at P15 and P30 were not apposed by PV^+^ terminals in the ventral MEC LII; however, we cannot exclude that there are also somata in the dorsal portion with no PV^+^ innervation. It is furthermore possible that the somata without PV^+^ terminal innervation in the cross-sections studied do in fact receive PV^+^ innervation at other levels.

### Major PV^+^ basket cell–related network changes before P15

Rats open their eyes at ∼14–15 days of age (Langston et al., 2010). From as early as P10, somata in dorsal and ventral parts of MEC LII formed symmetrical synapses with terminals containing PV immunoreactivity. In the period between P10 and P15, but not between P15 and P30, there was an increase in area of cross-sectional somata and amount of PV^+^ terminal apposition, displayed as an increased percentage of cross-sectional somata innervated by PV^+^ terminals and number and density of PV^+^ terminals per cross-sectional soma. The latter variable, which took into account the total length of PV^+^ terminal membrane apposed to a given cross-sectional soma, was not influenced by the displayed decrease in PV^+^ terminal cross-sectional area between P10 and P15, suggesting that the effective PV^+^ innervation of LII somata increases before eye opening. This increase could result from two very different developmental phenomena: on the one hand, a later emergence of PV protein expression in previously PV-immunonegative terminals (Seress and Ribak, 1990), and on the other hand, basket cell development to involve large increases in axonal length, axonal collateralizations, and convergence of presynaptic terminals, as other studies have found (Chattopadhyaya et al., 2004; Doischer et al., 2008; Hu et al., 2014). However, considering that PV^+^ terminals at P15 are smaller than at P10, it is very well possible that there is a shift that drives the replacement of initial large immature PV^+^ terminals at P10 with smaller and more numerous PV^+^ terminals at P15 (Portera-Cailliau et al., 2005).

Interestingly, our results of increased number of PV^+^ terminals before P15 are in contrast with data from the mouse primary visual cortex, where the number of PV^+^ perisomatic terminals was found to increase during a protracted period following eye opening, with a peak at P28 (Chattopadhyaya et al., 2004). As we found these changes to occur mostly before the onset of visual input, it suggests that PV^+^ basket cell–related maturation in MEC LII is largely influenced by intrinsically driven events, which is in keeping with a previous study on the maturation of the entorhinal-hippocampal circuit (Donato et al., 2017).

### Synaptic refinement during the emergence of grid cell activity

There is evidence that the smaller PV^+^ terminals observed at P15 are still immature (Couey et al., 2013). Stellate cells, the most numerous principal cell type in MEC LII (Gatome et al., 2010), are disynaptically connected through PV^+^ interneurons, and a previous study has demonstrated that functional inhibition between PV^+^ and stellate cells emerges at P16, after which there is an increase in recurrent inhibitory connections and amplitude of inhibition until adult-like levels are reached at P22 (Couey et al., 2013). This follows a similar timeline as the maturation of morphologic, intrinsic, and synaptic properties of hippocampal basket cells (Doischer et al., 2008) and emergence of stable grid cell activity in the rat MEC LII (Langston et al., 2010; Wills et al., 2010, 2012). Grid cells require both excitatory drive from the hippocampus indirectly through deep layers of MEC and direct PV^+^ inhibition to produce and maintain their hexagonal activity pattern (Bonnevie et al., 2013; Miao et al., 2017); thus, grid cell development is likely paralleled to the development of this presynaptic input. In dorsal and ventral MEC LII, we found no significant changes in cross-sectional area of somata or PV^+^ terminal number and density between P15 and P30, suggesting that the emergence of fast-spiking PV^+^ basket inhibition in this region is not related to such changes. There was, however, an increase in cross-sectional area of PV^+^ terminals. Whether terminal size is related to synaptic strength is not uniform, as both a positive correlation (Schikorski and Stevens, 1997; Mitchell et al., 2012) and no correlation (Branco et al., 2010) have been found between terminal volume and release probability.

In this respect, it is important to emphasize that the dorsal portion of MEC LII in one P30 animal displayed cells with, on average, a significantly lower number of PV^+^ terminals as well as PV^+^ terminals with larger cross-sectional area than the other P30 animal. This may be a result of the limitations in sampling PV^+^ terminals from thin sections. The results should be considered as approximative or semiquantitative, since the cells are not completely regular in shape or distribution around the soma (Chattopadhyaya et al., 2004; Ledoux and Woolley, 2005). It may also be due to an uneven sampling of cell types that are apposed by different numbers of PV^+^ perisomatic terminals (Armstrong et al., 2016). A third reason could be that somata in MEC LII in the two animals are differently innervated by PV^+^ terminals at P30, as the maturational process for the generation and elimination of synaptic contacts is highly dynamic (Portera-Cailliau et al., 2005).

Another aspect showing maturational changes between P15 and P30 was the percentage of dorsal and ventral PV^+^ terminals containing mitochondria, which increased from 30%–40% to 60%–70%. This percentage at P30 is similar to perisomatic inhibitory terminals innervating CA1 pyramidal neurons in the adult rat, where 76% were found to contain mitochondria (Ledoux and Woolley, 2005). Mitochondria in presynaptic terminals play several important roles, one of which is to mobilize vesicles from the reserve pool to replenish the readily release pool (Devine and Kittler, 2018). The size of the readily release pool is positively correlated with release probability (Dobrunz and Stevens, 1997; Murthy et al., 2001; Branco et al., 2010), and a study has shown that dynamin-related protein 1 (*Drp1*) knockout in flies, which leads to a reduced amount of terminal mitochondria, impairs synaptic transmission when stimulated at high frequency (Verstreken et al., 2005). A larger presence of mitochondria in PV^+^ terminals may therefore reflect an increased need for a machinery to support its fast-spiking characteristics necessary for proper grid cell activity.

### Dorsoventral differences in the development of PV^+^ basket cell synaptic circuitry

MEC LII displays gradients along the dorsoventral axis (DVA) in a number of ways: first, a physiologic larger grid field size and spacing and frequency of theta modulation (Brun et al., 2008); second, a reduction in size of stellate cells and increase in rise time and half-width of spontaneous excitatory postsynaptic potentials (Garden et al., 2008); third, a lower frequency of PV^+^ inhibitory inputs onto stellate cells and overall lower number of PV^+^ terminals (Beed et al., 2013); and fourth, a delayed development of the ventral part compared to the dorsal (Ray and Brecht, 2016; Donato et al., 2017). To investigate potential gradients in PV^+^ basket cell synaptic circuitry along the DVA, we compared PV^+^ terminals and target somata in the dorsal-most region of LII with the ventral-most region.

At all developmental stages, ventral somata were smaller and apposed by fewer PV^+^ terminals than in the dorsal portion. The morphology of dorsal and ventral PV^+^ terminals was, however, remarkably similar. Dorsoventral differences in soma size and amount of PV^+^ perisomatic innervation were present already at P10, suggesting that the development of the PV^+^ basket cell–related network is delayed in the ventral compared to the dorsal region, which is in close agreement with previous studies reporting a gradient of maturation along the DVA (Ray and Brecht, 2016; Donato et al., 2017). Based on colocalization of doublecortin, a marker for immature neurons, it was found that >80% of dorsal PV^+^ cells and ∼40% of ventral PV^+^ cells in mice are mature at P20 in MEC LII (Donato et al., 2017).

Larger somata and more numerous perisomatic PV^+^ terminals in dorsal LII are in keeping with studies reporting a gradient in size of stellate somata and number of PV^+^ terminals along the DVA (Garden et al., 2008; Beed et al., 2013). PV^+^ basket cells make up ∼60% of the total PV^+^ population; however, PV^+^ interneurons also comprise subtypes targeting the axon initial segments and distal dendrites of neurons. Moreover, PV^+^ basket cells target proximal dendrites as well as somata (Klausberger et al., 2003; Baude et al., 2007). Our results therefore indicate that the decrease in PV^+^ terminal number is at least partly due to fewer perisomatic PV^+^ terminals. Interestingly, it was found that dorsal stellate cells receive inhibition from PV^+^ cells that are both proximal, closer than 100 μm, and distal, >100 μm away. In contrast, ventral stellate cells are mainly targeted by proximal PV^+^ cells (Beed et al., 2013). This could imply that the larger number of perisomatic terminals on dorsal somata is not a result of a larger number of terminals originating from single PV^+^ cells, but rather a larger number of distinct PV^+^ cells innervating dorsal somata. Furthermore, our results indicate that PV^+^ terminals in the ventral portion of LII at P30 are on average smaller than in the dorsal portion. If the number of active zones scales up with terminal size (Schikorski and Stevens, 1997), it is possible that the release probability of dorsal PV^+^ perisomatic terminals is greater than ventral ones.

We cannot exclude that there are structural changes in LII somata and perisomatic PV^+^ terminals after P30, or that the reported dorsoventral differences are lost in the adult. A previous study on PV immunoreactivity in adult rats found up to six PV^+^ terminals per soma per thin section in MEC LII (Wouterlood et al., 1995), whereas we found up to 15 at P30. However, an absence of significant changes in cross-sectional soma area and amount of PV^+^ innervation between P15 and P30 suggest that these structural features stabilize during this time period, such that the dorsoventral differences in soma size and amount of PV^+^ innervation is maintained in the mature MEC LII. This is in line with the decreased size of stellate somata found along the DVA in the adult mouse (Garden et al., 2008).

### Functional relevance

Inhibitory inputs from fast-spiking PV^+^ cells have been found to be critical for the maintenance of the spatial modulation of grid cells (Buetfering et al., 2014; Miao et al., 2017), which is present from P16 onwards (Langston et al., 2010; Wills et al., 2010, 2012). Our findings suggest that grid cell activity emergence is linked to a strengthening of PV^+^ terminals by increased terminal size and presence of mitochondria after soma size and number and density of apposing PV^+^ terminals have stabilized. Furthermore, our data indicate that ventral somata are smaller and apposed by fewer and smaller PV^+^ terminals compared with dorsal somata at P30. This suggests that there is a gradient in the organization of MEC LII microcircuits along the DVA, which is in accordance with the decreased PV^+^ inhibition and theta modulation as well as increased grid field size and spacing toward the ventral part of LII (Brun et al., 2008; Beed et al., 2013).

## Synthesis

Reviewing Editor: Christian Lüscher, University of Geneva Decisions are customarily a result of the Reviewing Editor and the peer reviewers coming together and discussing their recommendations until a consensus is reached. When revisions are invited, a fact-based synthesis statement explaining their decision and outlining what is needed to prepare a revision will be listed below. The following reviewer(s) agreed to reveal their identity: Moritz Helmstaedter.

The referees raised a number of points, with which I concur and where I would like the authors to find appropriate answers.

1. The reporting of the data is in my view too parcelated. The key effect that the authors report is shown in figure 5 with a change in the density of parvalbumin terminals on somata, while with the underlying original data is distributed to two additional figures 4 and 3. These three numerical analyses should be combined because if the reviewer understands it correctly it is essentially dividing the number of parvalbumin terminals (figure 4) by the soma area (figure 3).

2. It would be very helpful if the presented data could be extrapolated to assumed soma sizes. When the authors for instance report “soma area” in figure 3 but this is in fact cross-sectional soma area in a very thin slice and not the surface area of a cell body, this is rather confusing. Also in order to be able to interpret these numbers in the context of circuit data would be very helpful to report extrapolated bouton numbers per actual cell body .

3. There seems to be a rather substantial mismatch between the weight of the reported data and the extended discussion. While this data is solid and relevant the multi-page discussion on functional implications of basket cells and inhibition in general in the medial entorhinal cortex seems a little bit over-scaled by this reviewer's opinion.

4. One aspect of the finding seems to be a conundrum: the authors report in addition to an increased density of synapses on somata also a decrease in synapse size. Since at least for excitatory terminals this size of synapses is assumed to be correlated with strength, such a decrease could potentially counteract the increased synapse density on the somata. As far as I can see this has not been discussed or quantitatively assessed in the manuscript? One solution would be to combine these numbers and report the effective somatic innervation area over age. This at least would give an indication whether these effects compensate or not.

5. the choice of parameters to be analyzed such as the aspect ratio is insufficiently motivated. Please cite an article that documents this aspect ratio as a relevant marker for PV cell development. Additionally, please consider the quantification of e.g. number of active zones, which has a direct relation to the functionality of the PV+ synapse.

6. Active zones: What are the criteria applied to identify an active zone? Were the number of vesicles close to the membrane taken into account? What is the number of active zone per bouton and what would be the evolution of the number of active zone on PV terminals with development?

7. Fig 2: ventral area, P15, left image of high magnification (500nm image): this image seems not to be centered onto the PV terminal shown by the right arrow on the image above (the 5um image). Make sure the image is centered on the terminal chosen.

8. What was the 24h-light-dark cycle imposed to the used rats? Were they all perfused at the same moment of the day? If not, how this would influence the results presented?

9. an important aspect of the development is the enrichment of PV-terminals around somata, however an equally important question would be to identify the proportion of cells not receiving PV input accross early developmental periods.

10. To further corroborate their studies it would be important that the authors compare with other studies. For example in Garden et coll. Neuron 2008, the authors measure (in a different way) similar values to the one presented in figure 3 and table 1 about soma size. In particular, the rats in the paper presented are young adults (P30), how the findings relate to more adult mice?

11. What would be the interpretation of the evolution of the dorso-ventral gradient in adulthood? Will the different parameters measured in the ventral area of MEC at P30 will continue to evolve in adulthood and reach similar level to the dorsal portion?

12. There are other documented parameters for PV-cells development, for example the axon diameter, which allow to infer about the reliability to propagate action potentials (Doisher et al J Neurosc. 2008, Hu, Gan & Jonas 2014). Based on the dataset available are there any statements that could be made?

13. Discussion l357: please further develop the possible role known from the literature of other types of interneurons.

14. The organization of stellate and pyramidal is very specific in layer 2 of MEC: pyramidal cells in lay 2/3 are located in patches islands surrounded by stellate cells. Can the author distinguished how different would be the perisomatic organization of PV terminals onto stellate and pyramidal cells?

Minor:

• Fig1a and Fig1b not cited in text (only Fig 1)

• Fig 1a and 1b: missing scale bar. Provide stereotactic coordinated if possible (particularly laterality of parasagittal section).

• Fig 2 and Fig 6: increase stroke of scale bar + use the same for all of them.

• Fig 2 arrows: On the low magnification image: label arrows in a way it is easily visible which arrow correspond to the higher magnification image below (color code or other code).

• Fig2: help readability of the figure by identifying soma on the higher magnification images.

• Fig 6 arrows: the arrows indicate synapse. However synapse size is not the same on all images, would it be possible to identify the limits of the active zones?

• Fig 3, Fig4, Fig 5, Fig 7, Fig 8: All axis are hardly visible, and some vertical Y axis are not visible. Make sure they have the same stroke and increase stroke.

• Check references, some have some misspelling: (ex l 465 Wills et al & Donato et al “80-”), or missing pages (Witter et al 2017)

Vocabulary:

• L137: “pre and postsynaptic membrane specializations”: can this terminology be further developed and specified?

• L178: “diminished”: it seems to me as a tendency to increase, Can this term be replaced for something similar to: “did not significantly increase same fold”.

• L385: please replace the vague term “more or less”.

Statistics:

A few elements would improve the statistics: (1) give result of mean comparison test (such as the ANOVA), (2) give exact p value when p value reached 0, (3) please repeat which statistical test is being used in each statistic information parenthesis in a consistent manner.

## Author Response

Dear editor,

Herewith I resubmit the following manuscript. We have made the requested changes in the figures for the bar-histograms. We have also made appropriate additional changes in the legends and in the description in the text. I expect you can approve the figure and textual changes.

Best regards, Johannes van der Want
